# PSYCHOSOCIAL RISK, SUBCLINICAL CVD, AND BRAIN VOLUMES: EXAMINING SEX DISPARITIES IN DEMENTIA RISK

**DOI:** 10.1093/geroni/igad104.2541

**Published:** 2023-12-21

**Authors:** Regina Wright, Desiree Bygrave

**Affiliations:** University of Delaware, Newark, Delaware, United States; Benedict College, Columbia, South Carolina, United States

## Abstract

The majority of the sex disparity in dementia remains unexplained. One potential risk factor for women’s higher incidence of dementia is subclinical cardiovascular disease (SCVD), including carotid atherosclerosis and arterial stiffness. To date, there is a significant gap in what is known about sex differences in the impact of psychosocial risk on pre-dementia risk factors both directly and via SCVD. Therefore, this study aimed to examine the mediating influence of SCVD on the relationship between psychosocial risk and brain volumes, as a proxy for pre-dementia risk, and whether the relationship is moderated by sex. The analysis included 136 older adults with a mean age of 68.04y who underwent neuropsychological, psychosocial, and vascular assessment (pulse wave velocity [PWV], carotid intima media thickness [IMT]), and 3T cranial magnetic resonance imaging. Moderated mediation analyses, adjusted for age and education, showed a mediating role of PWV in the relation of loneliness to total brain volumes (global measure of atrophy) was more pronounced among women (average causal mediation effect [ACME]=.44; p=.11). Unadjusted analyses trended toward moderated mediation, such that the mediating role of carotid IMT in the relation of anxiety to total WMLV (ACME=.001; p=.06) and total frontal WML volumes (ACME=.001; p=.05) was more pronounced among women than men. Findings suggest that in women, but not men, psychosocial risk factors may influence brain volumes via subclinical CVD. Additional research is needed to determine if these interrelations help to explain some of the sex disparity in dementia risk.

